# Osteolytic Breast Cancer Causes Skeletal Muscle Weakness in an Immunocompetent Syngeneic Mouse Model

**DOI:** 10.3389/fendo.2017.00358

**Published:** 2017-12-19

**Authors:** Jenna N. Regan, Carter Mikesell, Steven Reiken, Haifang Xu, Andrew R. Marks, Khalid S. Mohammad, Theresa A. Guise, David L. Waning

**Affiliations:** ^1^Department of Medicine, Indiana University School of Medicine, Indianapolis, IN, United States; ^2^Department of Physiology and Cellular Biophysics, Columbia University College of Physicians and Surgeons, New York, United States; ^3^Department of Cellular and Molecular Physiology, College of Medicine, Pennsylvania State University, Hershey, PA, United States

**Keywords:** breast cancer, osteolytic disease, muscle weakness, immune competent, syngeneic tumor model

## Abstract

Muscle weakness and cachexia are significant paraneoplastic syndromes of many advanced cancers. Osteolytic bone metastases are common in advanced breast cancer and are a major contributor to decreased survival, performance, and quality of life for patients. Pathologic fracture caused by osteolytic cancer in bone (OCIB) leads to a significant (32%) increased risk of death compared to patients without fracture. Since muscle weakness is linked to risk of falls which are a major cause of fracture, we have investigated skeletal muscle response to OCIB. Here, we show that a syngeneic mouse model of OCIB (4T1 mammary tumor cells) leads to cachexia and skeletal muscle weakness associated with oxidation of the ryanodine receptor and calcium (Ca^2+^) release channel (RyR1). Muscle atrophy follows known pathways *via* both myostatin signaling and expression of muscle-specific ubiquitin ligases, atrogin-1 and MuRF1. We have identified a mechanism for skeletal muscle weakness due to increased oxidative stress on RyR1 *via* NAPDH oxidases [NADPH oxidase 2 (Nox2) and NADPH oxidase 4 (Nox4)]. In addition, SMAD3 phosphorylation is higher in muscle from tumor-bearing mice, a critical step in the intracellular signaling pathway that transmits TGFβ signaling to the nucleus. This is the first time that skeletal muscle weakness has been described in a syngeneic model of OCIB and represents a unique model system in which to study cachexia and changes in skeletal muscle.

## Introduction

Breast cancer is the most common cancer in women (1) and frequently metastasizes to bone in advanced disease (2). Healthy bone has endocrine functions that are achieved both by active signaling from bone cells such as osteoblasts and osteocytes as well as passive release of cytokines and minerals stored in the bone matrix. However, in the tumor–bone microenvironment, breast cancer cells secrete factors that stimulate osteoclast-mediated bone resorption. The increased resorption, in turn, greatly increases the release of signaling factors from the mineralized matrix, including TGFβ. This further promotes cancer cell invasion, growth, and osteolytic factor production to fuel a feed-forward vicious cycle that induces more bone destruction (3–6). Increased bone resorption also causes skeletal complications such as bone pain, fractures, hypercalcemia, and nerve compression syndromes (6).

Skeletal muscle weakness is a debilitating consequence of many advanced malignancies. Muscle is one of the organ systems responsive to bone-derived signals. Thus, conditions such as osteolytic cancer in bone (OCIB) that disrupt the balance of normal bone resorption (7, 8) may also have detrimental effects on skeletal muscle. We have recently shown that a significant reduction in skeletal muscle function occurs in mice with osteolytic bone metastases from breast, prostate, and lung cancer and in multiple myeloma (9).

In normal muscle contraction, the ryanodine receptor calcium (Ca^2+^) release channel (RyR1) is activated, leading to the release of Ca^2+^ from the sarcoplasmic reticulum (SR) and causing muscle contraction. Ca^2+^ is then pumped back into the SR during relaxation by the sarcoplasmic/endoplasmic reticulum Ca^2+^ ATPase. Physiological oxidation is a normal signaling mediator in skeletal muscle whereas pathological oxidation of RyR1 leads to channel Ca^2+^ leak and muscle weakness (10, 11). We have previously shown that RyR1 oxidation and loss of its stabilizing subunit, calstabin1 (also known as FKBP12), is a biochemical signature of RyR1 channel Ca^2+^ leak in OCIB (9). This biochemical signature was also present in skeletal muscle samples taken from patients with breast cancer that had metastasized to bone, validating the clinical importance of our model systems. NADPH oxidase 4 (Nox4), a constitutively active oxidase and TGFβ target gene, is the source of reactive oxygen species in our models of OCIB that lead to skeletal muscle weakness. These data collectively describe a novel TGFβ-Nox4-RyR1 axis responsible for skeletal muscle weakness in OCIB (9).

The studies described above used human tumor cells, which made it necessary to perform the experiments in immunocompromised mice. Thus, we wondered whether the presence of a functional immune system would alter the TGFβ-Nox4-RyR1 axis that we identified. The present study addresses this question using a syngeneic murine model of breast cancer that is osteolytic in bone (4T1 cells). We have found that mice with 4T1 OCIB develop profound skeletal muscle weakness and cachexia within 4 weeks of tumor cell inoculation to bone. SMAD3 phosphorylation, the relative expression of Nox4 mRNA, and Nox4-RyR1 binding were higher in muscle from mice with 4T1 OCIB, consistent with disruption of the TGFβ-Nox4-RyR1 axis (9). In addition, skeletal muscle fiber diameter was reduced, and mRNA expression of the atrophy-related muscle-specific ubiquitin ligases, atrogin-1 and MuRF1, were higher in mice with OCIB than non-tumor control animals. The relative expression of myostatin mRNA, a strong mediator of muscle atrophy, was also higher in mice with OCIB. These data indicate that a syngeneic model of OCIB shows both muscle weakness due to Ca^2+^ mishandling and activation of a muscle atrophy program.

## Results

### Weight Loss in Mice with 4T1 OCIB

**Figure 1:**
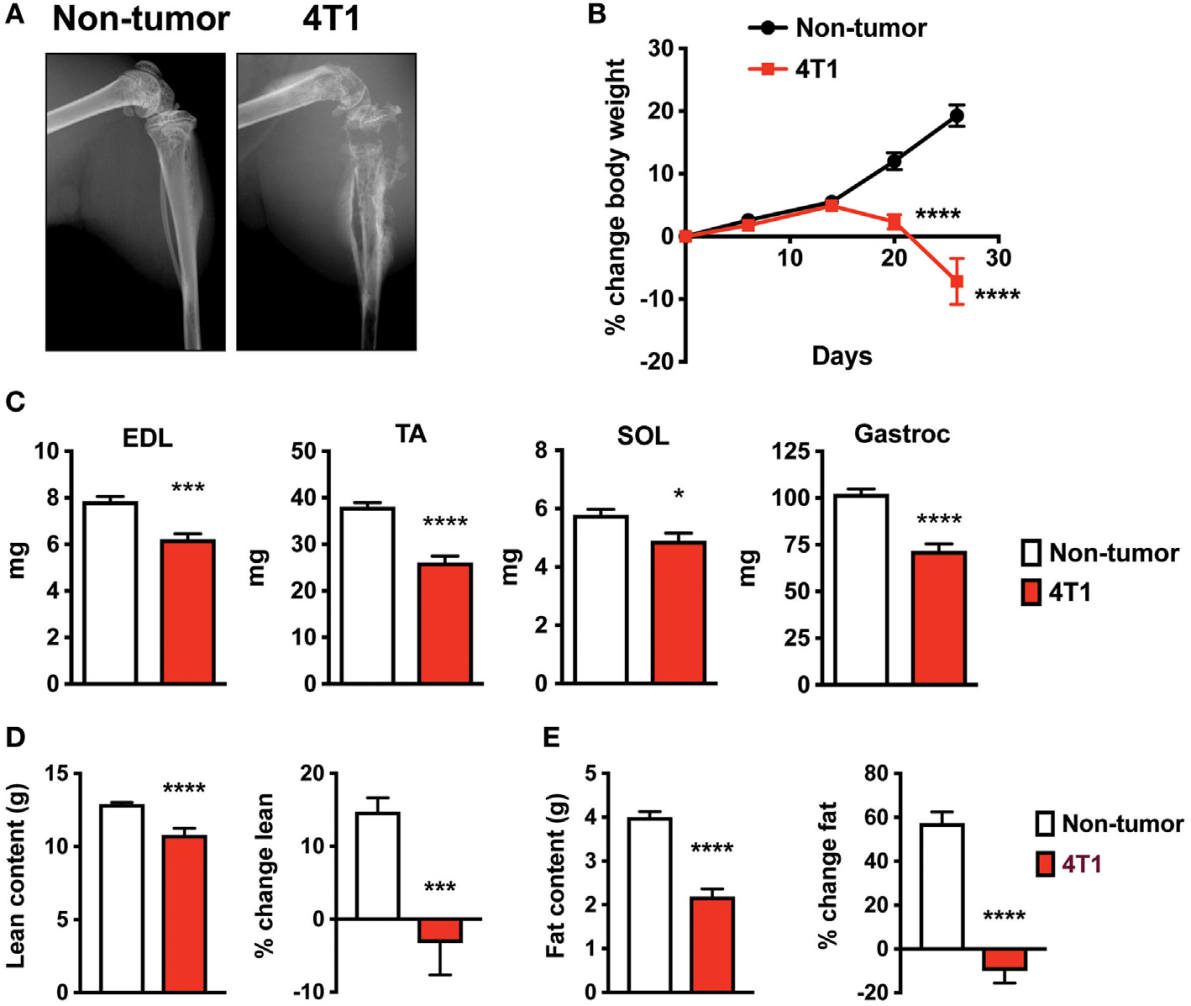
Mouse breast cancer 4T1 osteolytic cancer in bone (OCIB) leads to osteolysis and body weight loss. **(A)** X-ray analysis revealed large osteolytic lesions in mice receiving 4T1 cell *via* intratibial injection at 4-week postinjection. **(B)** Body weight loss in mice with 4T1 OCIB (percentage change in total body weight over baseline) (*n* = 10). **(C)** Weight loss of individual muscles dissected from the lower hindlimb (*n* = 10) of mice with 4T1 OCIB; extensor digitorum longus (EDL), tibialis anterior (TA), soleus (SOL), and gastrocnemius (Gastroc) compared to non-tumor control mice. Dual energy X-ray absorptiometry (DXA) scan revealed loss of both **(D)** lean content and **(E)** fat content in mice with 4T1 OCIB at 4-week postinjection (*n* = 10). Data are mean ± SEM, **(B)** two-way analysis of variance with Bonferroni post-test; **(C–E)** *t*-test. *p < 0.05, ***p < 0.001, and ****p < 0.0001.

To investigate skeletal muscle changes in an immune competent model of OCIB, we used 4T1 mouse stage IV breast cancer cells (12). We inoculated 100,000 cells (non-tumor mice received PBS), directly into the right tibia of 5-week old female Balb/c mice. Mice with 4T1 cells in bone exhibit osteolytic lesions as detected by X-ray (13–15). In our study, we confirmed this result at 4-week postinoculation (Figure 1A). We also found that mice with 4T1 OCIB had progressive weight loss starting at approximately 14-day postinoculation (Figure 1B). At 4-week postinoculation, mice with 4T1 OCIB had lower individual skeletal muscle weights measured following dissection. The extensor digitorum longus (EDL), tibialis anterior (TA), soleus (SOL), and gastrocnemius (Gastroc) muscles were dissected from hindlimb contralateral to the site of inoculation and weighed intact (Figure 1C). Mice with 4T1 OCIB also had lower lean (Figure 1D) and fat (Figure 1E) content as measured by dual energy X-ray absorptiometry (DXA). Lean and fat mass was measured both as absolute value at 4-week postinoculation and as the percentage change over the baseline reading (Figures 1D,E).

### 4T1 Osteolytic Bone Lesions Cause Skeletal Muscle Atrophy

**Figure 2:**
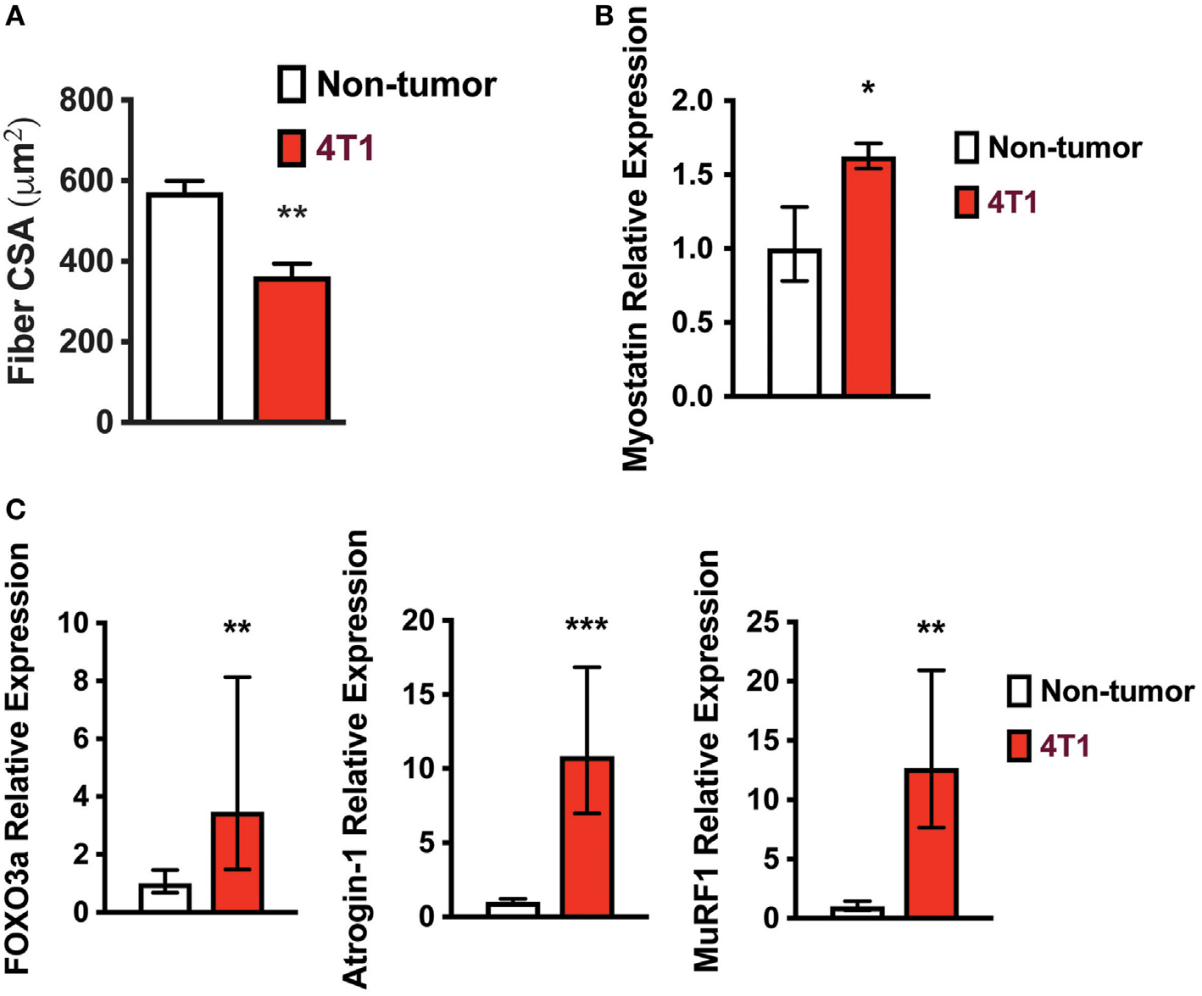
Skeletal muscle fiber size is lower in mice with 4T1 osteolytic cancer in bone (OCIB). **(A)** Gastrocnemius muscle fiber diameter was lower in mice with 4T1 OCIB (*n* = 3 histological sections) compared to non-tumor control mice. **(B)** Relative myostatin mRNA expression in tibialis anterior (TA) muscle from non-tumor mice and mice with 4T1 OCIB (*n* = 6). **(C)** Relative FOXO3, atrogin-1, and MuRF1 mRNA expression in TA muscle from non-tumor mice and mice with 4T1 OCIB (*n* = 6). **(A)** Data are mean ± SEM, error bars in **(B,C)** represent ddCt ± SD propagated to fold change, **(A–C)** *t*-test. *p < 0.05, **p < 0.01, and ***p < 0.001.

Because mice with 4T1 OCIB lost lean mass, we investigated known mechanisms of skeletal muscle atrophy. Skeletal muscle fiber cross-sectional area was lower in mice with 4T1 OCIB than in non-tumor control mice (Figure 2A). We measured cross-sectional area from H&E stained histological sections from the Gastroc muscle dissected from the side contralateral to tumor cell inoculation. At least 200 fibers were measured from histological sections taken from three samples from each group. Mice with 4T1 OCIB also had higher relative mRNA expression of myostatin, a strong negative regulator of skeletal muscle mass (16), compared to non-tumor controls (Figure 2B). In addition to myostatin, we also measured the relative expression of FOXO3a and its downstream targets atrogin-1 and MuRF1. The muscle-specific ubiquitin ligases atrogin-1 and MuRF1 are induced by FOXO3a by two distinct mechanisms during skeletal muscle atrophy (17–19). We found that the relative mRNA expression of FOXO3a, atrogin-1, and MuRF1 were all increased in mice with 4T1 OCIB compared to non-tumor control mice (Figure 2C).

### Skeletal Muscle Weakness in Mice with 4T1 OCIB

**Figure 3:**
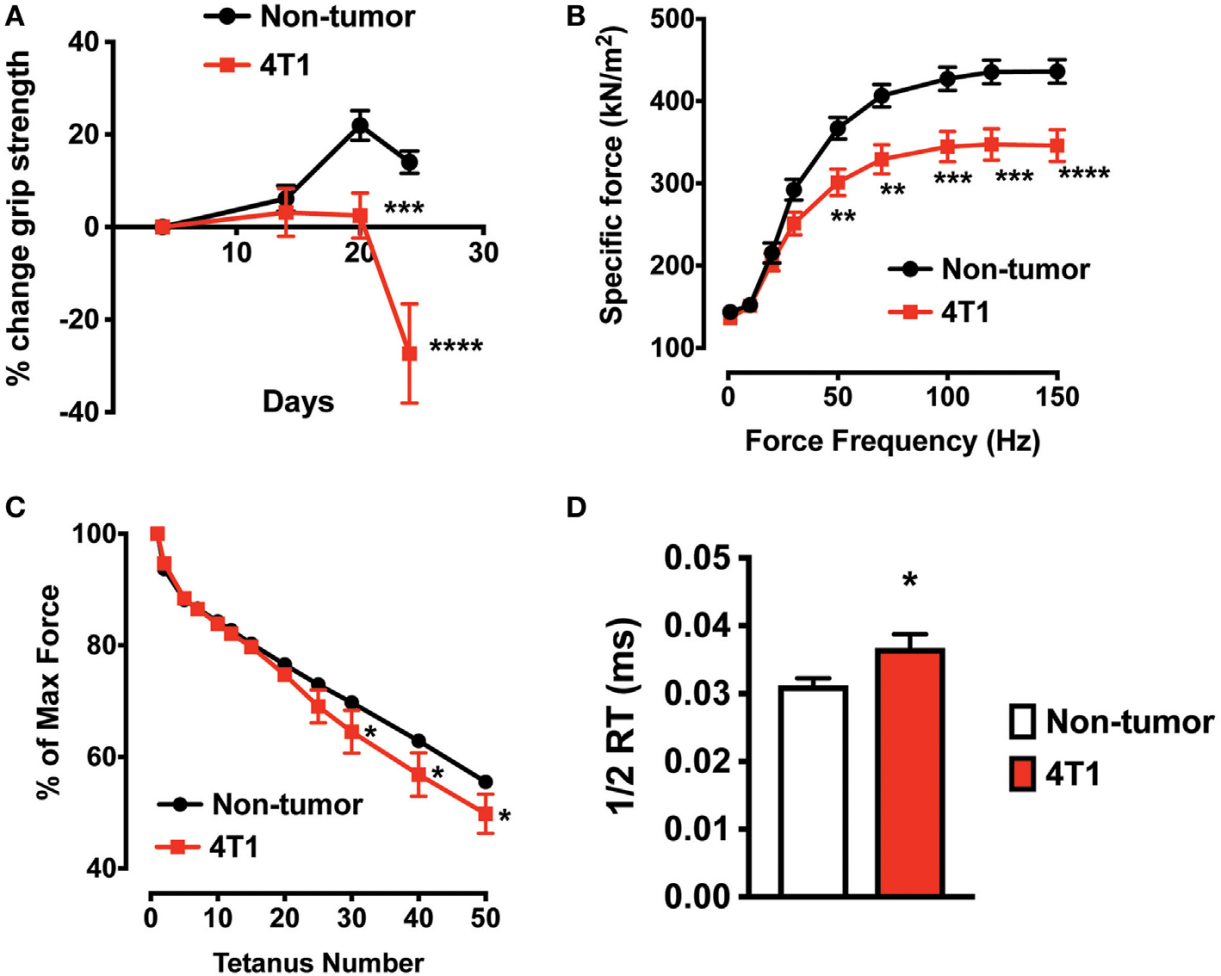
Skeletal muscle weakness in mice with 4T1 osteolytic cancer in bone (OCIB). **(A)** *In vivo* forelimb grip strength (*n* = 10) and **(B)** excised whole muscle contractility of the extensor digitorum longus (EDL) muscle (*n* = 10) were lower in mice with 4T1 OCIB. **(C)** Skeletal muscle fatigue was higher in mice with 4T1 cells in bone (percent of maximum force) (*n* = 10). **(D)** Half-relaxation time (1/2 RT) was higher in EDL from mice with 4T1 OCIB compared to non-tumor control mice. Data are mean ± SEM, **(A,B,C)** two-way analysis of variance with Bonferroni post-test, **(D)** *t*-test. *p < 0.05, **p < 0.01, ***p < 0.001, and ****p < 0.0001.

To determine skeletal muscle function in mice with 4T1 OCIB, we measured *in vivo* forelimb grip strength over the course of the experiment and at 4-week postinoculation we measured whole muscle contractility of the excised EDL muscle. Mice with 4T1 OCIB developed muscle weakness measured by forelimb grip strength starting at approximately 3-week postinoculation (Figure 3A). Whole muscle contractility of the excised EDL muscle showed lower muscle-specific force in tumor-bearing mice compared to non-tumor control animals (Figure 3B). Specific force corrects for differences in muscle size between individual animals and test groups (20). We also observed higher EDL fatigability in mice with 4T1 OCIB. Fatigue was tested by repeated whole muscle tetanic stimulation (Figure 3C). Finally, from our whole muscle contractility studies we determined the half-relaxation time of twitch stimulation of the EDL from mice with 4T1 OCIB. Tumor mice had a slower relaxation time compared to non-tumor control mice (Figure 3D) indicating dysfunctional Ca^2+^ handling in muscle.

### RyR1 Oxidation in Skeletal Muscle of Mice with 4T1 OCIB

**Figure 4:**
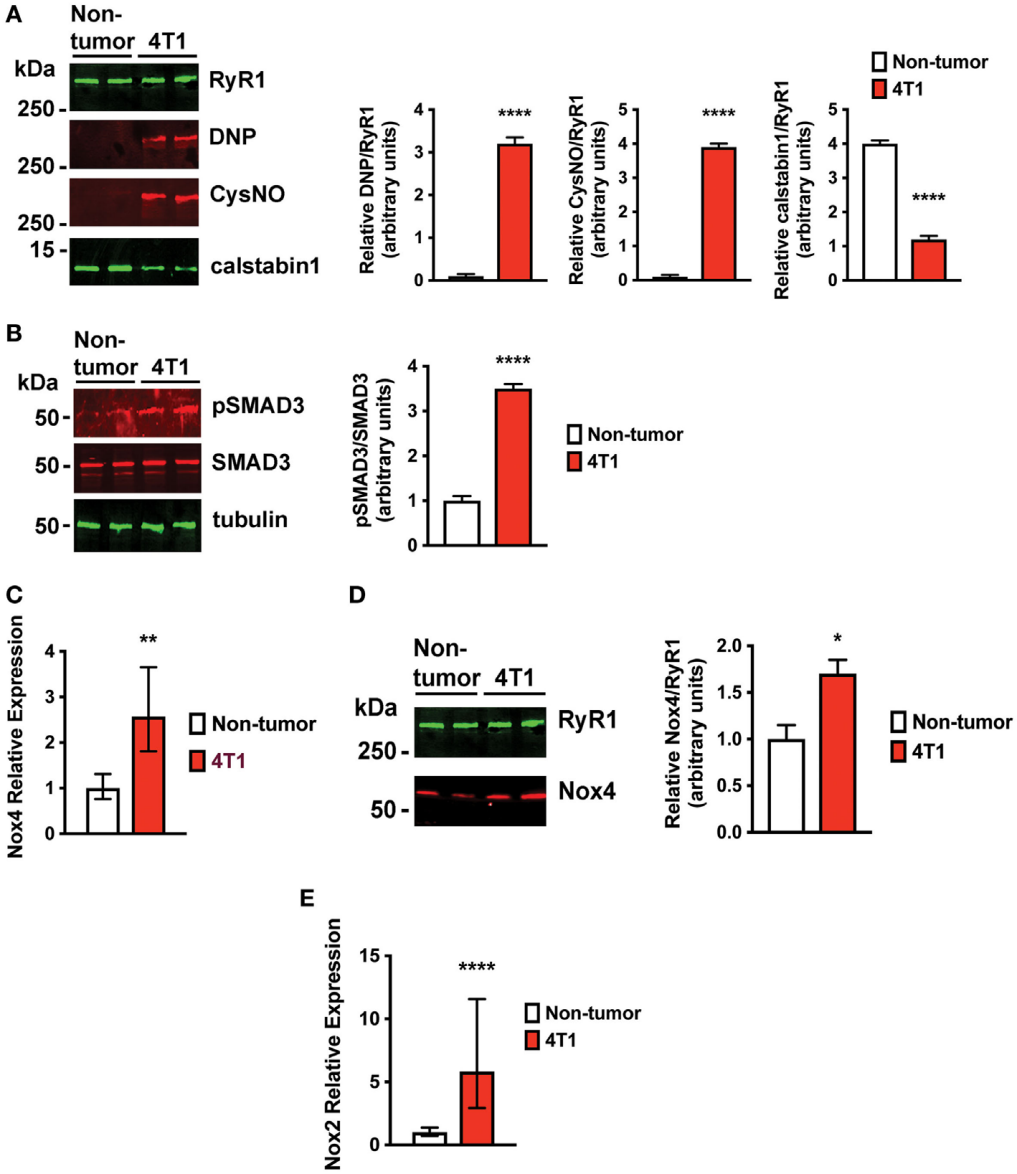
Skeletal muscle ryanodine receptor oxidation and SMAD3 phosphorylation are higher in mice with 4T1 osteolytic cancer in bone (OCIB). **(A)** Immunoblot (left) and quantification (right) of RyR1 oxidation (DNP) and nitrosylation (CysNO) and of RyR1-calstabin1 binding, as measured by coimmunoprecipitation, from extensor digitorum longus (EDL) muscle (*n* = 4). RyR1 was oxidized and depleted of calstabin1 in mice with 4T1 OCIB. **(B)** Immunoblot of SMAD3 phosphorylation (left) and quantification (right) from muscle revealed increased phosphorylation in mice with 4T1 OCIB compared to non-tumor control mice (*n* = 4). **(C)** Relative NADPH oxidase 4 (Nox4) mRNA expression was higher in tibialis anterior (TA) muscle from mice with 4T1 OCIB than in non-tumor control mice (*n* = 6). **(D)** Immunoblot (left) and quantification (right) of Nox4 coimmunoprecipitation with RyR1 from EDL muscle revealed higher Nox4 interaction with RyR1 in muscle from mice with 4T1 OCIB (*n* = 4). **(E)** Relative NADPH oxidase 2 (Nox2) mRNA expression was higher in TA muscle from mice with 4T1 OCIB than in non-tumor control mice (*n* = 6). **(A,B,D)** Data are mean ± SEM, error bars in **(C,E)** represent ddCt ± SD propagated to fold-change, **(A,B,D)** *t*-test. *p < 0.05, **p < 0.01, and ****p < 0.0001.

Increased oxidative stress is a characteristic of advanced breast cancer (21). Oxidation of RyR1 channels and reduced binding of the stabilizing subunit, calstabin1, in skeletal muscle result in pathological SR Ca^2+^ leak that is associated with muscle weakness (9–11). Indeed, skeletal muscle RyR1 channels from mice with 4T1 OCIB were oxidized, nitrosylated, and depleted of calstabin compared to non-tumor control mice (Figure 4A). We have previously shown that a major source of oxidative stress in OCIB is expression of Nox4 (9), a constitutively active Nox protein. Nox4 is a TGFβ target gene that is upregulated following the release of TGFβ from the bone matrix during OCIB (9). Mice with 4T1 OCIB showed higher SMAD3 phosphorylation, indicative of TGFβ signaling, compared to non-tumor control mice (Figure 4B). We also found higher relative mRNA expression of Nox4 and higher levels of direct Nox4-RyR1 binding compared to non-tumor control mice (Figures 4C,D). Our data also indicate an upregulation of NADPH oxidase 2 (Nox2) (Figure 4E) which could serve as another source of oxidative stress in muscle from mice with 4T1 OCIB. Nox2 and Nox4 are both induced in response to TGFβ in certain cell types (22), but interestingly, we did not observe upregulation of Nox2 mRNA in muscle in our previous studies using human tumor cells in athymic nude mice (9). The regulation of Nox2 gene expression in muscle represents a difference between immunocompetent and immunosuppressed mouse models of OCIB. Nox2 is thought to predominantly function in phagocytes (23) and activity of these cells in athymic nude mice may actually be enhanced (24). Thus, the role of Nox2 and Nox4 in OCIB-induced oxidative stress still needs to be determined.

## Discussion

Muscle weakness and cachexia are significant paraneoplastic syndromes of many advanced cancers. In the present study, we have investigated skeletal muscle weakness in a syngeneic mouse model of OCIB. Primary mouse 4T1 breast cancers are highly metastatic, including an affinity for bone, where the cells are aggressively osteolytic (14). Inoculating 4T1 tumor cells directly into the bone recapitulates the bone destruction (Figure 1A), while limiting metastasis to other organs and allowing investigation of bone to muscle signaling in the setting of OCIB. While we did observe metastases of 4T1 cells to lung, the number of cells inoculated and the time postinoculation were optimized for assessment of skeletal muscle changes in response to osteolytic lesion development. Importantly, this syngeneic system allows for the study of pathways important for changes in both muscle mass and muscle function in animals with an intact immune system and mimics the bone destruction and bone–muscle crosstalk that occur in humans with breast cancer bone metastases (9).

We have previously shown that TGFβ released from the bone matrix during osteolysis due to breast cancer bone metastases causes oxidative stress and skeletal muscle Ca^2+^ leak and weakness *via* the TGFβ-Nox4-RyR1 axis (9). This mechanism was elucidated in athymic nude mice to accommodate the use of human breast cancer cells (MDA-MB-231, ZR-75-1, and MCF-7), human lung cancer cells (RWGT2 and A549), human prostate cancer cells (PC-3), and human multiple myeloma cells (JJN-3). In addition, mice with MDA-MB-231, MCF-7, A549, or PC-3 bone metastases also develop cachexia that is independent of the reduction in skeletal muscle function.

While our previous studies strongly implicate bone-to-muscle signaling as the root cause of the skeletal muscle weakness, we felt it was important to investigate whether the presence of a functional immune system significantly alters OCIB-driven muscle weakness since this represents a major difference between the mouse models and human patients. In the present study, we have shown that mice with 4T1 OCIB develop both cachexia and skeletal muscle weakness. Myostatin expression and activation of the ubiquitin ligases atrogin-1 and MuRF1 downstream of FOXO3a are hallmarks of skeletal muscle atrophy (16–19) and are likely responsible for the cachexia in the 4T1 OCIB model. In addition, we determined that these immune competent mice display the same biochemical signature of RyR1 oxidation leading to skeletal muscle SR Ca^2+^ leak as immunodeficient mice with human tumor cells (9). The TGFβ signal mediator SMAD3 is phosphorylated in mice with 4T1 OCIB and that the relative expression of Nox4 mRNA and Nox4-RyR1 binding is higher than in non-tumor control mice. Overall, these data recapitulate those described in athymic nude mice and suggest that immune functionality does not significantly alter OCIB-induced cachexia or muscle weakness.

## Materials and Methods

### Animals

Female Balb/c mice were obtained from Harlan (Indianapolis, IN, USA) at 5 weeks of age. All experiments with animals were performed at the Indiana University School of Medicine (IUSM) and approved by Indiana University’s Institutional Animal Care and Use Committee (IACUC).

### Ethics Statement

In all studies, mice were handled and euthanized in accordance with approved institutional and national guidelines set forth by the Indiana University IACUC and the Laboratory Animal Resource Center at the IUSM. This facility is fully accredited by the American Association for the Accreditation of Laboratory Animal Care, International and is registered with the USDA as a Research Facility.

### Materials

Antibodies: Anti-RyR (Affinity Bioreagents, cat. no. MA3-916, Golden, CO, USA; 1:2,000), anti-RyR1 5029 (custom rabbit polyclonal antibody raised against the C-terminus of human RyR1, Yenzyme, South San Francisco, CA, USA; 1:250 for IP), anti-Cys NO antibody (Sigma, cat. no. N0409, St. Louis, MO, USA; 1:2,000), anti-calstabin antibody (Santa Cruz Biotechnology, cat. no. sc-6173, Santa Cruz, CA, USA, 1:2,500), anti-DNP (Oxyblot, cat. no. S7150, Millipore, Darmstadt, Germany; 1:250), anti-pSMAD3 (Abcam, cat. no. ab40854, Cambridge, UK; 1:1,000), anti-SMAD3 (Abcam, cat. no. 52903, Cambridge, UK; 1:1,1000), and anti-Nox4 (Abcam, cat. no. 109225, Cambridge, UK; 1:1,000).

### Cell Culture

4T1 breast cancer cells (ATCC, CRL-2539, Manassas, VA, USA) were cultured in Dulbecco’s modified Eagle’s media (Hyclone, Logan, UT, USA) containing 10% heat-inactivated fetal bovine serum. Cells were maintained at 37°C with 5% CO_2_ in a humidified chamber.

### *In Vivo* Models

Intratibial inoculation of tumor cells was performed on 5-week old female Balb/c mice. Tumor cells were trypsinized, washed twice in sterile ice cold PBS, and resuspended in ice cold PBS to a final concentration of 10^5^ cells in 20 µl. Mice were anesthetized (ketamine and xylazine) and inoculated in the proximal tibia using a 27-gauge needle. 100,000 cells (or PBS for non-tumor controls) were inoculated into the right tibia of each animal. Measurements of muscle weight and muscle function were done using the hindlimb contralateral to the side of tumor cell inoculation.

### Radiography

The presence of osteolytic lesions was visualized by radiography on a Kubtec digital X-ray imager (Kubtec, Milford, CT, USA). Mice were placed in a prone position and imaged at 2.7× magnification. The investigators were blinded to treatment subjects.

### Dual Energy X-ray Absorptiometry

Body composition (lean and fat content) was determined using a PIXImus mouse densitometer (GE Lunar II, Faxitron Corp., Tucson, AZ, USA). Mice were anesthetized and placed with limbs spread on an adhesive tray in a prone position. Measurements were performed and analyzed excluding the calvarium, mandible, and teeth. Values for lean and fat content were expressed as both a final absolute value and as a percentage change over the baseline scan. The investigators were blinded to treatment of subjects.

### Grip Strength

Forelimb grip strength was assessed by allowing each mouse to grab a wire mesh attached to a force transducer (Bioseb, Vitrolles, France) that records the peak force generated as the mouse is pulled by the tail horizontally away from the mesh. The investigators were blinded to treatment of subjects.

### Muscle Function

Whole muscle contractility of the EDL muscles was determined as previously described (9). EDL was dissected from hind limbs, and stainless steel hooks were tied to the tendons of the muscles using 4-0 silk sutures, and the muscles were mounted between a force transducer (Aurora Scientific, Aurora, ON, Canada) and an adjustable hook. The muscles were immersed in a stimulation chamber containing O_2_/CO_2_ (95/5%) bubbled Tyrode solution (121 mM NaCl, 5.0 mM KCl, 1.8 mM CaCl_2_, 0.5 mM MgCl_2_, 0.4 mM NaH_2_PO_4_, 24 mM NaHCO_3_, 0.1 mM EDTA, and 5.5 mM glucose). The force–frequency relationships were determined by stimulation of 0.5 ms pulses at 1–150 Hz for 350 ms with rest for 3 min between stimulations. Fatigue of the muscle was determined by calculating the percentage of maximum force generated at repeated tetanic stimulations. To quantify the specific force, the absolute force was normalized to the muscle size (20). The half-relaxation time was determined during muscle stimulations during twitch stimulation. The investigators were blinded to treatment of subjects.

### RyR1 Immunoprecipitation and Immunoblotting

RyR1 oxidation and nitrosylation and calstabin1 binding were determined from EDL muscles as previously described (9). Immunoblots were developed and quantified using the Odyssey Infrared Imaging System (LI-COR Biosciences, Lincoln, NE, USA) and infrared-labeled secondary antibodies. Detection of Nox4 (Abcam, Cambridge, UK; 1:1,000) was from immunoprecipitated RyR1 as described above. Detection of pSMAD3, SMAD3 (Abcam, Cambridge, UK; 1:1,000 each), and tubulin (Sigma, St. Louis, MO, USA; 1:500 each) from mouse muscle was *via* lysis in NP-40 buffer, and detection and quantification of immobilized proteins using the Odyssey Infrared Imaging System. The investigators were blinded to treatment of subjects.

### Semi-Quantitative RT-PCR

Tibialis anterior muscle was lysed by dounce homogenization in Trizol (Invitrogen) for RNA extraction. One-fifth volume chloroform was added to lysates and vortexed vigorously for 15 s and incubated at room temperature for 3 min. Samples were loaded onto GenElute mammalian total RNA mini columns (Sigma). DNase I treatment was performed to remove genomic DNA contamination (Qiagen), and RNA integrity was assessed on agarose gels. RNA was reverse transcribed using Superscript II (Invitrogen) with anchored oligo(dT) (Promega) for priming. The cDNAs were prepared for semiquantitative real-time PCR using HotStart-IT SYBR Green PCR Kit (Affymetrix) and analyzed in a CFX96 Real-Time PCR Detection System (BioRad) for 40 cycles (95°C for 15 s, 58°C 30 s, and 72°C for 30°s) after an initial 2 min incubation at 95°C. Target gene expression (Nox2 and Nox4), FOXO3a, atrogin-1, MuRF1, and myostatin were normalized against the housekeeping gene β2-microglobulin (B2M), and data were analyzed using the ΔΔCt method. Primers: B2M forward: 5′-CTG ACC GGC CTG TAT GCT AT-3′; B2M reverse 5′-CAG TCT CAG TGG GGG TGA AT-3′; Nox2 forward 5′-CCC TTT GGT ACA GCC AGT GAA GAT-3′; Nox2 reverse 5′-CAA TCC CGG CTC CCA CTA ACA TCA-3′; Nox4 forward 5′-GGA TCA CAG AAG GTC CCT AGC AG-3′; Nox4 reverse 5′-GCG GCT ACA TGC ACA CCT GAG AA-3′; FOXO3 forward 5′-CAG GCT CCT CAC TGT ATT CAG CTA-3′; FOXO3 reverse 5′-CAT TGA ACA TGT CCA GGT CCA A-3′, atrogin-1 forward 5′-GCA GAG AGT CGG CAA GTC-3′, atrogin-1 reverse 5′-CAG GTC GGT GAT CGT GAG-3′, MuRF1 forward 5′- GCT GGT GGA AAA CAT CAT TGA CAT-3′, and MuRF1 reverse 5′-ACT GGA GCA CTC CTG CTT GTA GAT-3′.

### Statistical Analysis

The data are presented as mean ± SEM. Differences among experimental groups were analyzed by *t-*tests or analysis of variance (ANOVA) with appropriate *post hoc* and multiple comparison tests. For single time point measures of any sample size, a two-sided *t*-test was used (25). When more than two groups were compared simultaneously ANOVA, followed by Bonferroni *post hoc* tests, was used (e.g., force–frequency comparison between control and tumor bearing groups). *p*-Values less than 0.05 were considered significant (*p < 0.05; **p < 0.01; ***p < 0.0005; and ****p < 0.0001). Statistical analyses were performed with Prism 6.0 software (GraphPad Prism, La Jolla, CA, USA). The number of mice required to assess muscle function in mice with OCIB was determined by power analysis using previous data on improvement in muscle function in mice with bone metastases. From the mean difference in specific force production with OCIB vs vehicle was 42% (275 vs 390 kN/m^2^) with SD = 64 kN/m^2^. Assuming α error rate = 0.05 and β error rate = 0.20, and a more conservative 30% mean difference (275–360), the minimum number of animals per group needed is *n* = 8 for a two-sided Student’s *t*-test. Based on the experience of the Investigators, approximately 80% of mice injected develop tumors; so 10 mice per group were used. Exclusion plan: EDL-specific force data were excluded in cases where there was evidence of damage to the muscle fibers. This exclusion plan was pre-established. Female Balb/c mice received from Harlan (Indianapolis, IN, USA) were randomized into groups upon arrival.

## Ethics Statement

In all studies, mice were handled and euthanized in accordance with approved institutional and national guidelines set forth by the Indiana University Institutional Animal Care and Use Committee and the Laboratory Animal Resource Center (LARC) at the Indiana University School of Medicine (IUSM). This facility is fully accredited by the American Association for the Accreditation of Laboratory Animal Care, International (AAALAC) and is registered with the USDA as a Research Facility.

## Author Contributions

All contributing authors have agreed to submission of this manuscript for publication. JR, AM, KM, TG, and DW conceived of the study. JR, AM, KM, TG, and DW designed experiments, analyzed data, and interpreted results. JR, KM, CM, SR, HX, and DW performed experiments. JR, KM, TG, and DW wrote the manuscript.

## Conflict of Interest Statement

The authors declare that the research was conducted in the absence of any commercial or financial relationships that could be construed as a potential conflict of interest.
